# Association between estimated plasma volume status and the risk of 30-day mortality in patients with severe acute pancreatitis: a retrospective study based on the MIMIC-IV database

**DOI:** 10.1186/s12876-025-03895-y

**Published:** 2025-04-29

**Authors:** Haibo Zhang, Jiebin Li, Dawei Wang, Jing Wang, Lijun Duan, Jing Zhang

**Affiliations:** Department of Emergency, Beijing Tongren Hospital, Capital Medical University, No. 1 Dong Jiao Min Xiang, Dong Cheng District, Beijing, 100730 China

**Keywords:** Acute pancreatitis, Estimated plasma volume status, 28-day mortality, MIMIC-IV database

## Abstract

**Background:**

Assessing plasma volume is important in the management and treatment of severe acute pancreatitis (SAP). Although it is an easy and rapid method for estimating the plasma volume, the association between estimated plasma volume status (ePVS) and the prognosis of SAP remains elusive. This study was aimed at assessing the relationship of ePVS with the risk of 30-day all-cause mortality (ACM) in SAP patients.

**Methods:**

This study collected clinical data on SAP patients in the ICU from the MIMIC-IV database. LASSO regression was used to screen for relevant covariates. The nonlinear relationship of ePVS with the risk of 30-day ACM was assessed utilizing the restricted cubic spline (RCS) analysis, and then their association was assessed by a multivariate Cox regression model. 30-day survival across different groups was compared by a Kaplan-Meier survival curve.

**Results:**

This study included 1036 patients, with a 30-day survival rate of 86.8%. They were assigned to four groups by quartiles of ePVS. The Kaplan-Meier survival curve showed that the high ePVS group was at a higher risk of 30-day ACM (*p* = 0.007). Multivariate Cox regression analysis showed a positive association of ePVS as a continuous variable with the risk of 30-day ACM (HR = 1.09, 1.01–1.18, *p* = 0.035). The risk of 30-day ACM was higher in the Q4 group vs. the Q1 group with ePVS as a categorical variable (HR = 1.70, 95% CI: 1.03–2.80, *p* = 0.039). RCS analysis showed a linear relationship of ePVS with the risk of 30-day ACM (*p* = 0.606), with a cut-off value of 6.23 dL/g. Subgroup analysis revealed significant associations between the two within specific subgroups, but ePVS did not interact with any of the subgroup variables.

**Conclusion:**

Our findings showed a significant association of high ePVS values with an increased risk of 30-day ACM. This study helps to identify high-risk patients early and guide the development of personalized treatment strategies.

**Supplementary Information:**

The online version contains supplementary material available at 10.1186/s12876-025-03895-y.

## Strengths and limitations of this study

- This study used data from the Mimic database to ensure consistency and reliability through strict data cleaning and standardization processing.
- The MIMIC-IV database and its accompanying Mimic Code Repository are open-source, ensuring comparability and reproducibility of our research.
- Few studies have discussed the correlation between plasma volume status estimation and 30 day mortality risk in patients with severe acute pancreatitis.
- Most of the patients in the study come from European and American countries. Whether these data match the physical characteristics of populations in other countries or regions remains to be debated.

**Supplementary Information:**

The online version contains supplementary material available at 10.1186/s12876-025-03895-y.

## Introduction

Acute pancreatitis (AP) is an inflammatory disorder of the pancreas. It is mainly caused by gallstone disease (40–65%) and alcohol abuse (25–40%). Its other causes include hypertriglyceridemia, hypercalcemia, familial disorders, viral infections, and periampullary tumors [1]. The incidence and hospitalization rates of AP tend to increase in Asia, Europe and the Americas [2]. Severe AP (SAP) can lead to serious complications with a mortality of 10–30 [3]. AP is histopathologically characterized by damaged secretory acinar cells caused by primary or secondary factors, which activates trypsinogen to inappropriately release trypsin and further activates other digestive enzymes, thereby resulting in pancreatic autolysis in the parenchyma [4]. The pathophysiologic mechanisms of SAP are not yet fully understood. Although AP can be diagnosed with the aid of relevant laboratory tests and imaging examinations and genetic studies have been published, there are still many difficulties and controversies in the etiologic diagnosis, treatment, and prognostic evaluation of AP. Its severity and prognosis are often evaluated using scoring systems such as the Acute Physiology and Chronic Health Evaluation (APACHE) II score, Bedside Index of Severity in Acute Pancreatitis (BISAP) score [5], and CT severity index [6]. However, the above scoring systems require a large number of vital signs and laboratory results or imaging findings as a basis for scoring. Simple and effective methods for evaluating the severity and prognosis of SAP are more convenient for clinical use.

The estimated plasma volume status (ePVS) formula, which evolved from the Strauss formula, can be calculated to assess plasma volume at a given time point [7]. Current studies on ePVS have found its correlation with functional impairment of the kidneys, lungs, heart and other organs, prolonged hospitalization, and an increased risk of death during the treatment of some serious diseases [8]. SAP is a complex and serious disease. There is still no consensus on the choice of moderate fluid resuscitation or aggressive fluid resuscitation as in early SAP [9]. Monitoring of plasma volume during the management of SAP may be helpful in evaluating its severity and prognosis. The accuracy of ePVS in evaluating the prognosis of SAP is little known.

This study intended to analyze the relationship of ePVS with the risk of 30-day ACM in SAP patients by collecting and analyzing relevant clinical data from the Medical Information Mart for Intensive Care IV (MIMIC-IV version 2.2), thereby helping evaluate the severity and prognosis of SAP.

## Methods

### Database

Data were collected from the above database (version 2.2) [10], which was created by Massachusetts Institute of Technology’s Laboratory of Computational Physiology. This database collected clinical data on patients hospitalized at the Beth IsraelDeaconess Medical Center between 2008 and 2019, including a large amount of data specific to the ICU. The author, Haibo Zhang, has completed a certification program and can access this database to extract data (Record ID: 63644403). To protect patient privacy, all ID information of patients was hidden.

### Criteria for population selection

Data on AP patients were searched using disease codes from the International Classification of Diseases, 9th Revision and 10th Revision, Clinical Modification (ICD-9-CM) and (ICD-10-CM) codes. Patients aged > 18 years and initially admitted to the ICU were included in this study. Exclusion criteria were as follows: Non-ICU patients, and patients with missing data on hematocrit or hemoglobin.

### Data extraction

Clinical data on patients included age, gender, marital status, race, and comorbidities including obesity and overweight, hypertension (HT), acute kidney injury (AKI), atrial fibrillation (AF), diabetes mellitu (DM), and heart failure. Scoring systems included the Sequential Organ Failure Assessment (SOFA) score, and Severe Acute Pancreatitis Score II (SAPS II). Vital signs included the heart rate (HR), systolic blood pressure (SBP), diastolic blood pressure (DBP), mean arterial pressure (MAP), respiratory rate (RR), pulse oxygen saturation (SPO2), and temperature. Laboratory results involved albumin, alkaline phosphatase (ALP), alanine aminotransferase (ALT), aspartate aminotransferase (AST), lactate dehydrogenase (LDH), total bilirubin (TBIL), blood urea nitrogen (BUN), chloride, creatinine, blood lipase, phosphate, glucose, potassium, sodium, total calcium, international normalized ratio (INR), lactate (Lac), anion gap (AG), bicarbonate, white blood cell (WBC) count, platelet (PLT) count, hematocrit (HCT), and hemoglobin (HBG). Treatment involved mechanical ventilation (MV), norepinephrine, statins, proton pump inhibitors (PPI), Qctreotide, hospital stay, and ICU stay. All results were the first test results after admission to the ICU. Both drugs and ventilation were started within 24 h after ICU admission.

### Handling of outliers and missing values

Variables with more than 20% of missing data were excluded. Variables with less than 20% of missing data were interpolated using a multiple imputation method. Outliers were processed using the Winsorization method to reduce the impact of outliers on data analysis, and the upper and lower limits were 99% and 1%, respectively.

### ePVS definition

ePVS was estimated using the Strauss formula, which considered HCT and the HBG concentration as estimates of PVS. The formula is as follows [11]:

ePVS (dL/g)=(100 − HCT((%))/HBG (g/dL).

### Outcome

The outcome of this study was the risk of 30-day ACM in SAP patients admitted to the ICU. The risk of 30-day ACM indicated death from any cause in patients within 30 days from the first day of hospitalization.

### Statistical analysis

Categorical variables were expressed as frequencies and percentages and analyzed using the Chi-square test. Continuous variables were expressed as the mean (standard deviation) and analyzed using the t-test if they followed a normal distribution confirmed by the Shapiro-Wilks test. Otherwise (if not), they were expressed as the median (interquartile range) and analyzed using nonparametric methods (Mann-Whitney U test). All confounders were analyzed by LASSO regression, and 10-fold cross-validation was performed to derive λ values and screen out prognosis-related covariates. The survival probability of each ePVS group was visualized using the Kaplan-Meier (KM) curve and compared using a log-rank test. The independent correlation between the ePVS level and the risk of 30-day ACM in AP patients was assessed using univariate and multivariate Cox regression analyses. Results were expressed as hazard ratios (HRs) with 95% confidence intervals (CIs). Model I was unadjusted. Age was adjusted in Model II. Model III was further adjusted for SBP, albumin, BUN, INR, LDH, blood lipase, total TBIL, AKI, DM, hyperlipidemia, norepinephrine use, and statin use. The relationship of ePVS with the risk of 30-day ACM was checked by RCS analysis. Subgroup analysis was performed by age ( > = 60 vs. <60), gender, marital status, race, AKI, AF, DM, heart failure, obesity and overweight, HT, ventilation, norepinephrine, statins, PPIs, and octreotide. Data were analyzed by R 4.4.0, and a 2-tailed *p*-value of < 0.05 was considered statistically significant.

## Results

### Demographics and baseline characteristics

A total of 1,036 patients were finally included in this study. They included 524 (50.6%) patients younger than 60 years of age, 583 (56.3%) males, 676 (65.3%) whites, and 446 (43.1%) married patients. The median length of ICU stay for all patients was 2.75 (1.73; 5.24) days. There were 899 (86.8%) 30-day survivors and 137 (13.2%) deaths. All included patients were assigned by quartiles of ePVS data (5.14 dL/g, 6.26 dL/g, 7.77 dL/g) to a Q1 group (*n* = 258), Q2 group (*n* = 260), Q3 group (*n* = 259), and Q4 group (*n* = 259). There were significant differences (< 0.05) among different ePVS groups in gender, AKI, obesity and overweight, SBP, DBP, MAP, SPO2, WBC count, hematocrit, hemoglobin, albumin, ALP, AST, ALT, LDH, blood lipase, phosphate, AG, BUN, INR, SAPS II, and SOFA. The ePVS group had lower levels of SBP, DBP, MAP, WBCs, hemoglobin, albumin, AST, ALT, LDH, blood lipase. AKI was more likely to occur in the high ePVS group. Obesity and overweight were more likely to be present in the low ePVS group. Detailed results are shown in Table 1.

**Table 1:** The characteristic of included subjects between different groups

Variable	Level	Overall *n* = 1,036	Q1 *n* = 258	Q2 *n* = 260	Q3 *n* = 259	Q4 *n* = 259	*p*-value
General information							
Age group (%)	< 60	524 (50.6%)	147 (57.0%)	130 (50.0%)	130 (50.2%)	117 (45.2%)	0.063
	>=60	512 (49.4%)	111 (43.0%)	130 (50.0%)	129 (49.8%)	142 (54.8%)	
Gender (%)	Female	453 (43.7%)	85 (32.9%)	107 (41.2%)	122 (47.1%)	139 (53.7%)	< 0.001
	Male	583 (56.3%)	173 (67.1%)	153 (58.8%)	137 (52.9%)	120 (46.3%)	
Marital status(%)	Single	341 (32.9%)	92 (35.7%)	83 (31.9%)	81 (31.3%)	85 (32.8%)	0.352
	Divorced/Widowed	185 (17.9%)	39 (15.1%)	47 (18.1%)	56 (21.6%)	43 (16.6%)	
	Married	446 (43.1%)	114 (44.2%)	111 (42.7%)	112 (43.2%)	109 (42.1%)	
	Unknow	64 (6.18%)	13 (5.04%)	19 (7.31%)	10 (3.86%)	22 (8.49%)	
Race(%)	White	676 (65.3%)	159 (61.6%)	171 (65.8%)	178 (68.7%)	168 (64.9%)	0.655
	No White	262 (25.3%)	71 (27.5%)	63 (24.2%)	63 (24.3%)	65 (25.1%)	
	Unknow	98 (9.46%)	28 (10.9%)	26 (10.0%)	18 (6.95%)	26 (10.0%)	
Comorbidities							
Obesity and overweight(%)	No	854 (82.4%)	196 (76.0%)	208 (80.0%)	224 (86.5%)	226 (87.3%)	0.001
	Yes	182 (17.6%)	62 (24.0%)	52 (20.0%)	35 (13.5%)	33 (12.7%)	
HT (%)	No	411 (39.7%)	94 (36.4%)	101 (38.8%)	110 (42.5%)	106 (40.9%)	0.528
	Yes	625 (60.3%)	164 (63.6%)	159 (61.2%)	149 (57.5%)	153 (59.1%)	
AKI(%)	No	444 (42.9%)	114 (44.2%)	121 (46.5%)	117 (45.2%)	92 (35.5%)	0.048
	Yes	592 (57.1%)	144 (55.8%)	139 (53.5%)	142 (54.8%)	167 (64.5%)	
AF(%)	No	770 (74.3%)	189 (73.3%)	198 (76.2%)	197 (76.1%)	186 (71.8%)	0.599
	Yes	266 (25.7%)	69 (26.7%)	62 (23.8%)	62 (23.9%)	73 (28.2%)	
DM(%)	No	597 (57.6%)	153 (59.3%)	138 (53.1%)	159 (61.4%)	147 (56.8%)	0.253
	Yes	439 (42.4%)	105 (40.7%)	122 (46.9%)	100 (38.6%)	112 (43.2%)	
Heartfailure:	No	752 (72.6%)	193 (74.8%)	188 (72.3%)	186 (71.8%)	185 (71.4%)	0.825
	Yes	284 (27.4%)	65 (25.2%)	72 (27.7%)	73 (28.2%)	74 (28.6%)	
Score system							
SOFA (median [IQR])		4.00 [2.00;7.00]	4.00 [2.00;7.00]	4.00 [2.00;6.00]	4.00 [2.00;7.00]	5.00 [3.00;8.00]	0.018
SAPSII (median [IQR])		33.0 [24.0;43.0]	31.0 [23.0;43.0]	30.5 [22.0;42.2]	34.0 [24.5;43.0]	36.0 [26.0;46.0]	0.006
Vital signs							
HR (median [IQR])		93.0 [80.0;109]	96.0 [81.0;114]	91.0 [80.0;104]	95.0 [80.0;111]	91.0 [79.0;107]	0.022
SBP (median [IQR])		126 [110;145]	137 [117;150]	126 [112;144]	123 [109;142]	121 [105;137]	< 0.001
DBP (median [IQR])		71.0 [59.0;83.0]	77.0 [67.0;93.0]	71.5 [60.8;83.0]	69.0 [56.5;79.0]	65.0 [53.0;76.5]	< 0.001
MAP (median [IQR])		84.0 [72.8;97.0]	92.0 [80.2;106]	85.5 [77.0;97.2]	82.0 [71.0;96.0]	78.0 [67.0;90.0]	< 0.001
HR (median [IQR])		93.0 [80.0;109]	96.0 [81.0;114]	91.0 [80.0;104]	95.0 [80.0;111]	91.0 [79.0;107]	0.022
RR (median [IQR])		19.0 [16.0;24.0]	19.0 [16.0;24.0]	19.0 [16.0;23.0]	19.0 [15.0;23.0]	20.0 [16.0;24.0]	0.158
SPO2 (median [IQR])		97.0 [95.0;99.0]	96.0 [94.0;98.0]	97.0 [95.0;99.0]	98.0 [95.5;100]	98.0 [96.0;100]	<0.001
Temperature (median [IQR])		98.2 [97.6;98.9]	98.3 [97.5;99.0]	98.2 [97.7;98.8]	98.3 [97.6;99.0]	98.2 [97.6;98.9]	0.781
Laboratory results							
Albumin (median [IQR])		3.10 [2.70;3.60]	3.30 [2.90;3.80]	3.30 [2.80;3.70]	3.10 [2.70;3.60]	2.90 [2.50;3.30]	<0.001
ALP (median [IQR])		90.5 [66.0;156]	81.5 [61.2;140]	96.0 [68.0;154]	101 [68.0;172]	93.0 [66.5;164]	0.014
ALT (median [IQR])		40.0 [20.0;96.0]	47.0 [24.2;134]	45.0 [23.8;114]	38.0 [20.0;102]	31.0 [15.5;60.0]	<0.001
AST (median [IQR])		52.5 [27.0;128]	58.0 [31.0;145]	62.0 [28.8;150]	49.0 [30.0;124]	42.0 [22.0;95.0]	<0.001
LDH (median [IQR])		273 [199;445]	320 [214;538]	274 [206;461]	249 [188;386]	263 [190;396]	<0.001
Tbil (median [IQR])		0.80 [0.50;2.10]	0.90 [0.50;2.10]	0.75 [0.48;1.90]	0.80 [0.40;2.30]	0.70 [0.40;2.20]	0.171
BUN (median [IQR])		19.0 [12.0;33.0]	19.0 [12.0;30.0]	17.0 [11.0;29.0]	18.0 [11.0;37.5]	23.0 [14.5;37.5]	0.001
Chloride (median [IQR])		105 [101;109]	105 [101;108]	104 [100;109]	106 [101;109]	105 [101;110]	0.185
Creatinine (median [IQR])		1.00 [0.70;1.60]	1.00 [0.70;1.50]	0.90 [0.70;1.50]	0.90 [0.70;1.70]	1.00 [0.70;2.00]	0.446
Bloodlipase (median [IQR])		93.0 [33.0;434]	190 [50.0;722]	93.0 [33.0;404]	67.0 [29.0;250]	83.0 [28.0;299]	<0.001
Phosphate (median [IQR])		3.30 [2.50;4.10]	3.20 [2.50;4.10]	3.20 [2.30;4.00]	3.20 [2.40;4.10]	3.70 [2.75;4.40]	<0.001
Glucose (median [IQR])		122 [99.0;162]	125 [103;182]	121 [97.0;160]	120 [97.0;156]	122 [98.0;154]	0.102
Potassium (median [IQR])		4.00 [3.60;4.40]	4.00 [3.62;4.47]	3.90 [3.60;4.30]	4.00 [3.60;4.40]	4.00 [3.60;4.50]	0.272
Sodium (median [IQR])		138 [136;141]	139 [136;141]	138 [136;141]	138 [136;141]	139 [135;141]	0.732
Totalcalcium (median [IQR])		8.10 [7.50;8.60]	8.20 [7.43;8.70]	8.20 [7.68;8.70]	8.10 [7.60;8.60]	7.90 [7.40;8.40]	0.003
INR (median [IQR])		1.30 [1.10;1.50]	1.20 [1.10;1.40]	1.20 [1.10;1.42]	1.30 [1.10;1.60]	1.30 [1.10;1.70]	<0.001
Lac (median [IQR])		1.70 [1.20;2.60]	1.90 [1.40;2.60]	1.70 [1.20;2.60]	1.70 [1.10;2.50]	1.70 [1.05;2.80]	0.33
AG (median [IQR])		14.0 [12.0;17.0]	15.0 [13.0;18.0]	14.0 [12.0;17.0]	15.0 [12.0;18.0]	14.0 [12.0;17.0]	0.003
Bicarbonate (median [IQR])		23.0 [19.0;25.2]	22.0 [19.0;26.0]	23.0 [20.0;26.0]	22.0 [18.0;25.0]	22.0 [19.0;25.0]	0.051
WBC (median [IQR])		10.4 [7.00;15.7]	11.9 [8.43;16.6]	10.4 [6.70;16.0]	10.0 [7.20;14.9]	9.00 [5.85;14.6]	< 0.001
PLT (median [IQR])		191 [128;273]	183 [135;240]	194 [136;271]	195 [131;278]	192 [116;306]	0.453
HCT (median [IQR])		32.5 [27.9;36.8]	40.3 [38.4;43.6]	34.5 [33.4;35.7]	30.2 [29.0;31.6]	25.6 [23.1;27.0]	< 0.001
HBG (median [IQR])		10.8 [9.30;12.3]	13.6 [12.8;14.5]	11.6 [11.2;12.0]	10.0 [9.70;10.4]	8.40 [7.70;8.90]	< 0.001
Treatment							
Ventilation (%)	No	580 (56.0%)	143 (55.4%)	149 (57.3%)	150 (57.9%)	138 (53.3%)	0.711
	Yes	456 (44.0%)	115 (44.6%)	111 (42.7%)	109 (42.1%)	121 (46.7%)	
Norepinephrine (%)	No	770 (74.3%)	189 (73.3%)	195 (75.0%)	191 (73.7%)	195 (75.3%)	0.943
	Yes	266 (25.7%)	69 (26.7%)	65 (25.0%)	68 (26.3%)	64 (24.7%)	
Statins (%)	No	650 (62.7%)	173 (67.1%)	162 (62.3%)	160 (61.8%)	155 (59.8%)	0.376
	Yes	386 (37.3%)	85 (32.9%)	98 (37.7%)	99 (38.2%)	104 (40.2%)	
PPI (%)	No	230 (22.2%)	62 (24.0%)	70 (26.9%)	57 (22.0%)	41 (15.8%)	0.019
	Yes	806 (77.8%)	196 (76.0%)	190 (73.1%)	202 (78.0%)	218 (84.2%)	
Qctreotide (%)	No	947 (91.4%)	243 (94.2%)	246 (94.6%)	231 (89.2%)	227 (87.6%)	0.007
	Yes	89 (8.59%)	15 (5.81%)	14 (5.38%)	28 (10.8%)	32 (12.4%)	
ICU los (median [IQR])		2.75 [1.73;5.24]	2.77 [1.72;5.97]	2.75 [1.72;4.75]	2.73 [1.73;4.79]	2.75 [1.76;4.82]	0.86
Status 30d:	Alive	899 (86.8%)	233 (90.3%)	235 (90.4%)	218 (84.2%)	213 (82.2%)	0.008
	Dead	137 (13.2%)	25 (9.69%)	25 (9.62%)	41 (15.8%)	46 (17.8%)	

Results are expressed as mean (SD), median [IQR] or n (%). DM, Diabetes Mellitus; HT, Hypertension; SOFA, Sequential Organ Failure Assessment score; Charlson, comorbidity index;

APACHE II, Acute Physiology And Chronic Health Evaluation II; SASPII, Severe Acute Pancreatitis Score II; HR, Heart Rate; SBP, Systolic Blood Pressure; DBP, Diastolic Blood Pressure;

MAP, Mean Arterial Pressure; RR, Respiratory Rate; ePVS, Estimated plasma volume status; Lac, Lactate; WBC, White Blood Cell count; PLT, Platelet count; HCT, hematocrit; ALT, Alanine Aminotransferase; AST, Aspartate Aminotransferase; PT, Prothrombin Time; INR, International Normalized Ratio; BUN, Blood Urea Nitrogen; Cr, Creatinine; CRRT, Continuous Renal Replacement Therapy; AKI, Acute Kidney Injury; Losicu, Length of ICU Stay

### Screening for covariates linked to the risk of 30-day ACM using LASSO regression

**Fig. 1:**
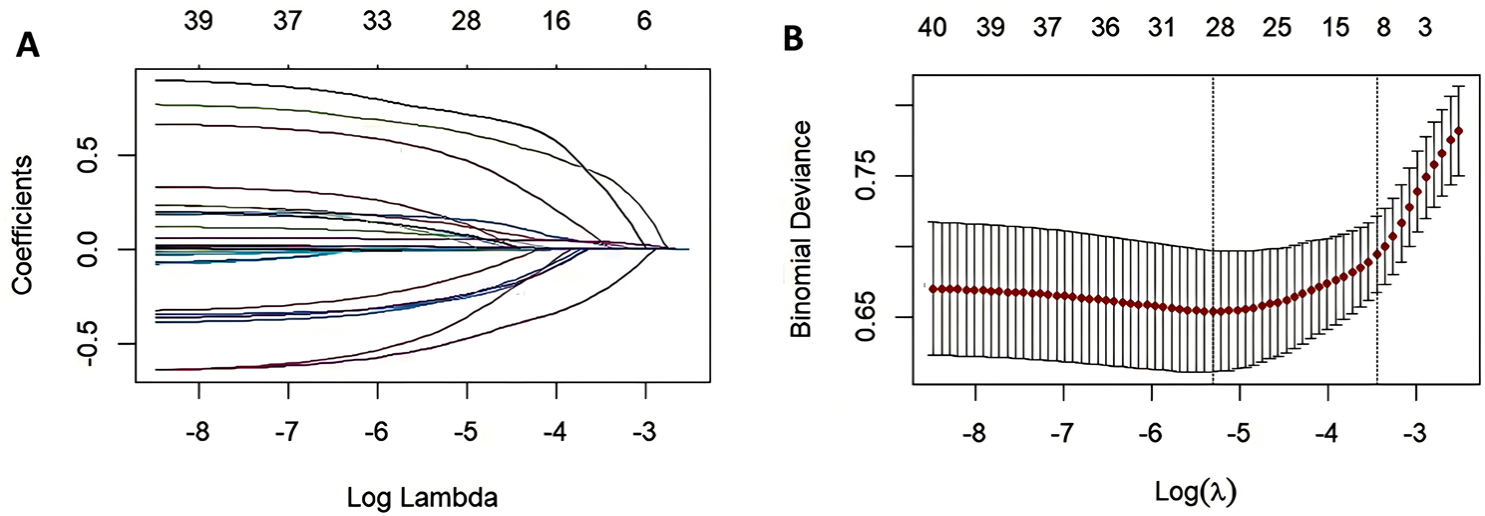
Lasso Regression. (**A**) Process of Selection variables by LASSO regression. (**B**) Process of selecting the parameter of best lambda value by cross-validation

All covariates were analyzed using LASSO regression analysis, and 10-fold cross-validation was performed to derive a value of 0.026 for the regularization parameter λ, as shown in Fig. 1. All covariates linked to the risk of 30-day ACM were identified, including age, SBP, albumin, BUN, INR, LDH, blood lipases, phosphate, TbIL, AKI, DM, hyperlipidemia, norepinephrine use, and statins use.

### KM survival curve

**Fig. 2:**
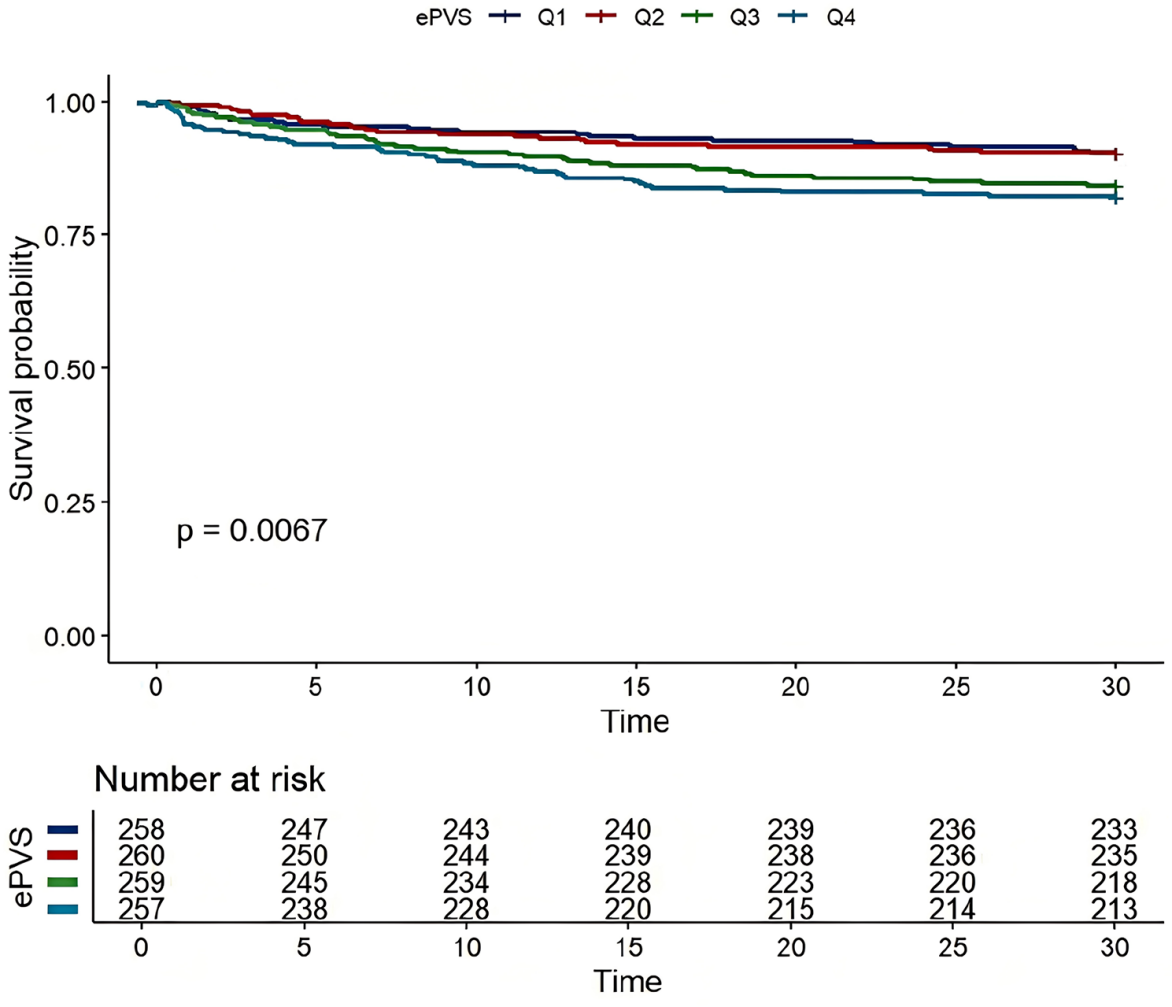
Kaplan–Meier survival curve of 30-day cumulative survival rate for every ePVS groups (Q1, Q2, Q3 and Q4)

The risk of 30-day ACM was significantly increased in the high ePVS group vs. the low ePVS group. Specifically, the mortality was 9.69% in the Q1 group, 9.62% in the Q2 group, 15.8% in the Q3 group, and highest (17.8%) in the Q4 group (*p* = 0.007). These data are presented via the KM survival curve (Fig. 2).

### COX regression for determining the association of ePVS with the risk of 30-day ACM

To determine the association of ePVS with the risk of 30-day ACM in SAP patients, we conducted Cox regression analysis, in which ePVS was included in the model as both continuous and categorical variables. First, we performed univariate Cox regression analysis with ePVS as a continuous variable. The results showed that ePVS was significantly positively associated with the risk of 30-day mortality in Model I (HR = 1.16, 95% CI: 1.07–1.26, *p* < 0.001). In Model II, ePVS was still significantly positively associated with the risk of mortality (HR = 1.14, 95% CI: 1.05–1.24, *p* < 0.001). In Model III, there was also an association of ePVS with such risk, as shown in Table 2.

**Table 2:** ePVS levels and all-cause 30 days mortality of acute pancreatitis

	Model I, HR 95%CI, *p* value	Model II, HR, 95%CI, *p* value	Model III, HR, 95%CI, *p* value
30-day mortality			
ePVS (continuous variable)	1.16 (1.07–1.26, *p* < 0.001)	1.14 (1.05–1.24, *p* < 0.001)	1.09 (1.01–1.18, *p* = 0.035)
ePVS [Categorical variables (quartile)]			
Q1	Ref	Ref	Ref
Q2	0.99 (0.57–1.73, *p* > 0.9)	0.93(0.54–1.63, *p* = 0.8)	0.98 (0.56–1.72, *p* > 0.9)
Q3	1.69(1.03–2.77, *p* = 0.04)	1.58(0.96–2.61, *p* = 0.07)	1.39(0.82–2.35, *p* = 0.2)
Q4	1.94 (1.19–3.16, *p* = 0.008)	1.74(1.07–2.84, *p* = 0.026)	1.70(1.03–2.80, *p* = 0.039)
P for trend	*p* = 0.001	*p* = 0.004	*p* = 0.015

Model I: No adjustments

Model II: Adjusted for Age

Model III: In addition to Model II adjustments, further adjustments were made for Systolic Blood Pressure (SBP), Albumin, Blood Urea Nitrogen (BUN), International Normalized Ratio (INR), Lactate Dehydrogenase (LDH), Bloodlipase, Total Bilirubin(Tbil), Acute Kidney Injury (AKI), Diabetes Mellitus (DM), Hyperlipidemia, Norepinephrine use, Statins use

Similarly, univariate and multivariate Cox regression analyses with ePVS as a categorical variable were done to determine the effects of different ePVS levels on the risk of 30-day ACM in SAP patients. In Model I, the ePVS level was positively associated with such risk in the high ePVS group vs. the low ePVS group (Q3: HR = 1.69, 95% CI, 1.03–2.77, *p* = 0.04; Q4: HR = 1.94, 95% CI, 1.19–3.16, *p* = 0.008; p for trend = 0.001). In Model II, there was still an association of a high ePVS level with a higher risk of 30-day ACM (Q3: HR = 1.58, 95% CI, 0.96–2.61, *p* = 0.07; Q4: HR = 1.74, 95% CI, 1.07–2.84, *p* = 0.026; p for trend = 0.004). In Model III, the risk of mortality was higher in the high ePVS group vs. the low ePVS group (Q4: HR = 1.70, 95% CI, 1.03–2.80, *p* = 0.039; p for trend = 0.015).

### RCS analysis for observing the nonlinear relationship of ePVS with the risk of 30-day ACM

**Fig. 3:**
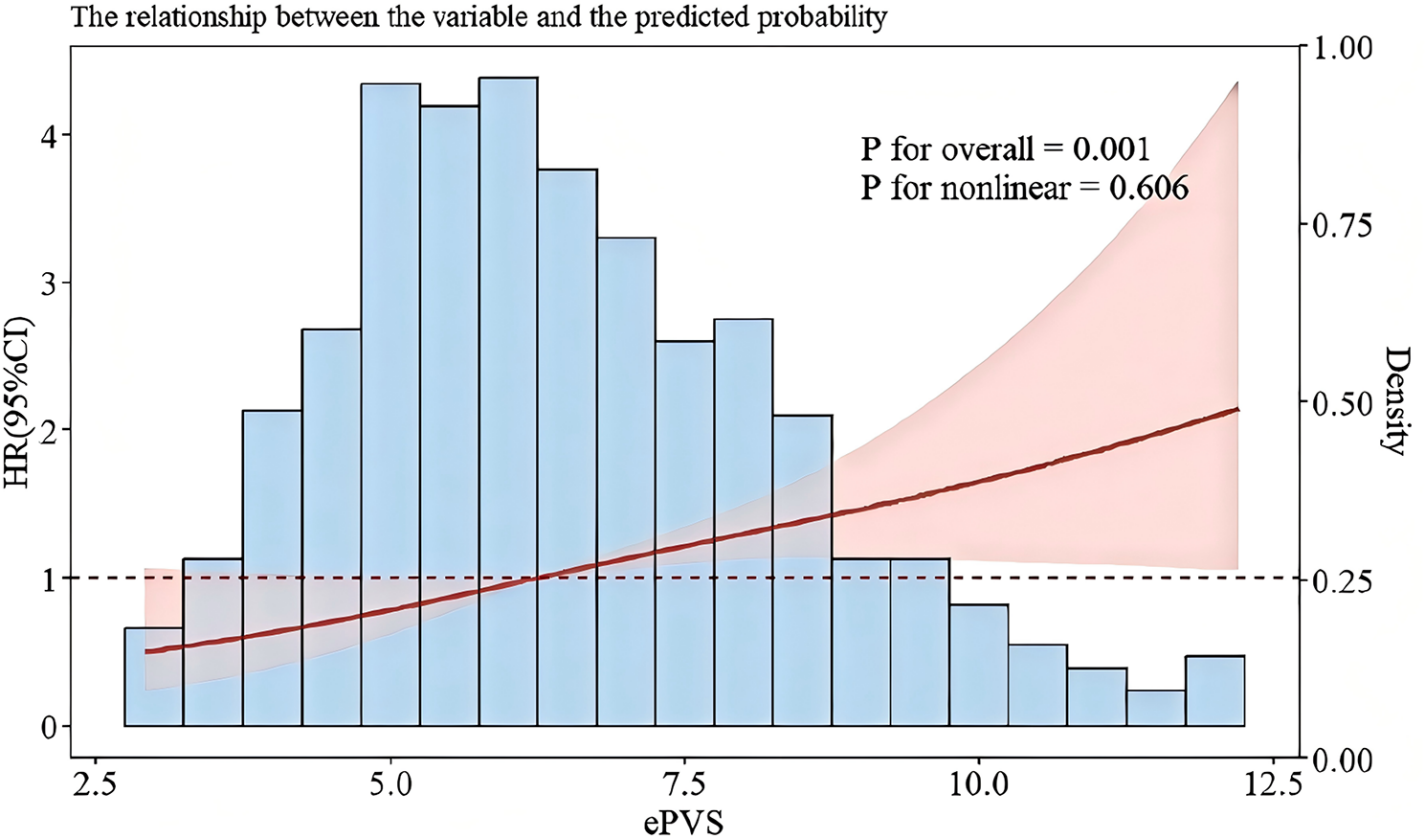
Restricted Cubic Spline (RCS) analyses of the correlation between estimated plasma volume status(ePVS)and the risk of 30-day all-cause mortality in patients with severe acute pancreatitis (SAP)

RCS analysis revealed a linear relationship between the level of ePVS at ICU admission and the risk of 30-day ACM (*p* = 0.606), as shown in Fig. 3. When the HR was 1, the cut-off value of ePVS was determined to be 6.23 dL/g.

### Subgroup analysis

**Fig. 4:**
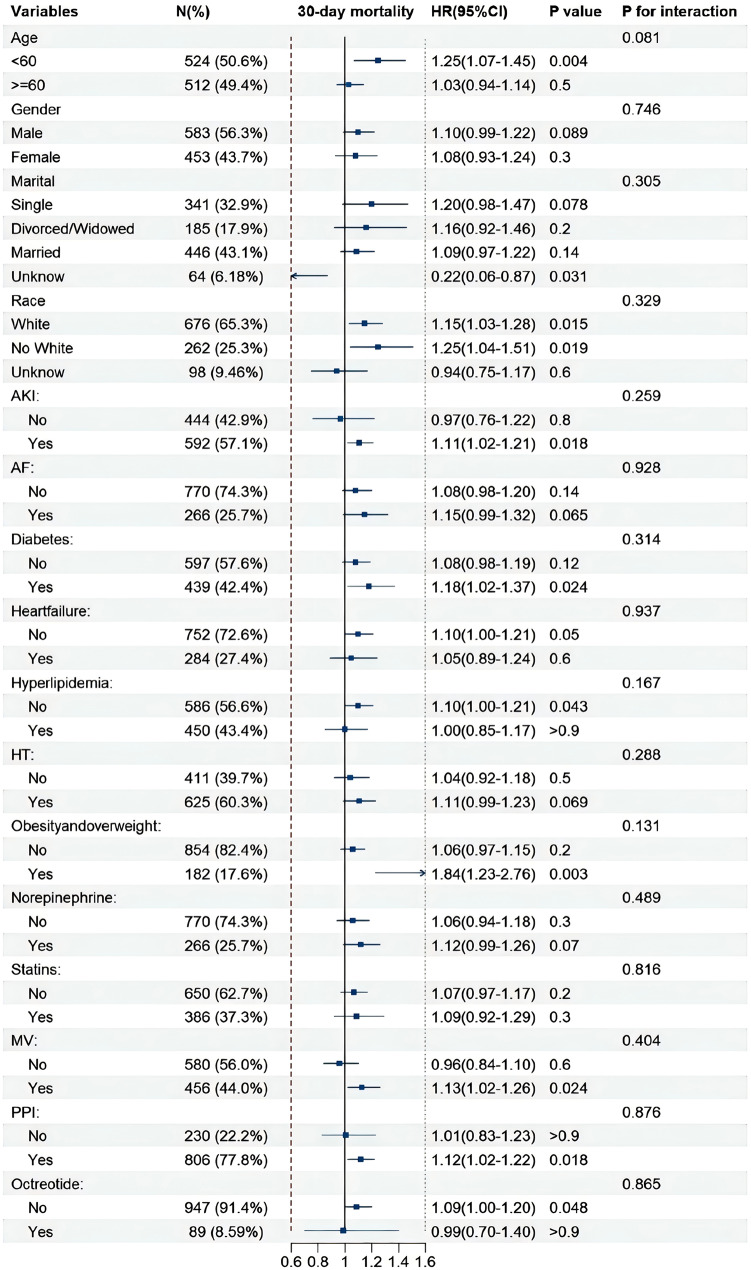
Subgroup analysis of the association between ePVS and 30-day mortality

Subgroup analyses were performed according to age, gender, marital status, race, AKI, AF, DM, heart failure, hyperlipidemia, HT, obesity and overweight, norepinephrine use, statins use, PPI use, octreotide use, and MV. Subgroup analysis revealed a significant association within specific subgroups, as shown in Fig. 4. The association of ePVS with the risk of 30-day ACM was more significant in patients younger than 60 years (HR = 1.25, 95% CI: 1.07–1.45), white patients (HR = 1.15, 95% CI: 1.03–1.28), patients experiencing AKI (HR = 1.11, 95% CI:1.02–1.21), patients with comorbid DM (HR = 1.18, 95% CI:1.02–1.37), obese/overweight patients (HR = 1.84, 95% CI = 1.23–2.76), patients using norepinephrine (HR = 1.12, 95% CI = 0.99–1.26), patients using MV (HR = 1.13, 95% CI = 1.06–1.26), and patients not using octreotide (HR = 1.09, 95% CI = 1-1.2). ePVS showed no interaction with all included subgroup variables, with all *p* values for interaction higher than 0.05.

## Discussion

MIMIC is a large clinical database that brings together high-quality clinical data on a wide range of ICU and emergency patients, which are continuously updated. Exhaustive clinical data on 1,036 SAP patients admitted to the ICU were collected from the MIMIC database in this study. To ensure its scientificity and reliability, the data were rigorously cleaned and screened to exclude variables with a high percentage of missing data and interpolate data with a low percentage of missing data. The results showed a total of 137 deaths (13.2%) within 30 days. The relationship of ePVS at ICU admission with the risk of 30-day ACM was explored using RCS analysis. It was found that the risk of 30-day ACM increased with ePVS before it reached the cut-off value of 6.23 dL/g. Interestingly, RCS analysis also indicated a linear relationship between the level of ePVS at ICU admission and the risk of 30-day ACM (*p* = 0.606, Fig. 3). This suggests that mortality risk will increase as ePVS exceeds the cut-off value, while the relationship will become less predictable with levels below such value. The KM curve also showed a significant difference in survival across different ePVS levels, and a lower survival rate was found in the high ePVS group. Cox regression permits to evaluate simultaneously the impact of multiple covariates (adjusted for) on survival. Therefore, univariate and multivariate Cox regression analyses were used in our study to identify independent predictors of 30-day ACM in ICU patients with SAP. The results of multivariate Cox regression analysis with ePVS as either a continuous or categorical variable showed a significantly higher risk of mortality in patients with a high ePVS level vs. those with a low ePVS level, indicating that there was a positive association of ePVS with the risk of mortality in SAP patients. In addition, our study found a significantly higher incidence of AKI (64.5%) in the high ePVS group (especially the Q4 group) vs. the other ePVS group, which may imply an association of ePVS with renal impairment.

Recent advancements in computational methods have underscored the growing importance of advanced analytical techniques in clinical research. For instance, deep learning applied to medical imaging, such as multi-modal MRI-based brain tumor segmentation [12], demonstrated the potential of sophisticated algorithms to enhance diagnostic precision and disease characterization [12]. Similarly, our study aligns with this trend by leveraging robust statistical methodologies, including RCS analysis and Cox regression models, to elucidate the relationship between ePVS and mortality in SAP. These statistical tools, aimed at generating feature-preserving networks for retinal vasculature segmentation, enable nuanced interpretation of complex clinical data, and help reveal critical thresholds (e.g., an ePVS cut-off value of 6.23 dL/g) and linear relationships (*p* = 0.606) for patient risk stratification [13].

Radioisotopes are the gold standard for assessing plasma volume. However, this method is costly, carries radiation risks, and is not available in most healthcare facilities. Given the clinical needs, several alternative methods have emerged to assess plasma volume, among which ePVS is a clinically accessible and rapid indicator for plasma volume. Initially, the Strauss formula ∆ePVS was proposed for assessing the change in plasma volume between two time points [14]. Subsequently, based on ∆ePVS, Duarte, et al. developed a formula for ePVS calculation, which was able to calculate instantaneous plasma volume using hematocrit and hemoglobin values at a single time point [15]. Clinical studies of ePVS have focused on cardiovascular disease (CVD), and numerous studies have confirmed an association of ePVS with a poor prognosis of CVD. For example, ePVS was positively linked to the risk of in-hospital mortality in patients with acute myocardial infarction with an area under the receiver operating characteristic (ROC) curve of 0.667 (95% CI: 0.653–0.681) [16]. In addition, the ePVS value in combination with the fractional excretion of urea nitrogen (FEUN) was significantly linked to the risk of ACM in patients with acute decompensated heart failure (HR, 2.92 [95% CI, 1.73–4.92; *P* < 0.001]) [9]. In those with pre-capillary pulmonary hypertension, ePVS was associated with the occurrence of cardiorenal syndrome and the survival rate during follow-up (HR, 2.33, 95% CI [1.49, 3.63]) [17]. The reasons for the association of ePVS with adverse cardiovascular events may be twofold. On the one hand, an increased cardiac load in a high plasma volume state leads to increased stasis in tissues/organs; on the other hand, the change in plasma volume state may increase the risk of thrombosis [18].

In the management of severe diseases such as SAP, accurately assessing plasma volume status in patients is crucial for guiding and timely adjusting treatment regimens. SAP at the early stage is frequently accompanied by systemic inflammatory response syndrome (SIRS), which leads to loss of fluid in the third interstitial space and transfer of intravascular fluid to the extracellular space, thereby causing tissue hypoperfusion. Hence, early fluid resuscitation is required [18]. During fluid resuscitation, it is critical to monitor plasma volume to avoid over-hydration, which may increase the risk of poor prognosis. In early fluid resuscitation in acute pancreatitis, aggressive intravenous rehydration (> 4.475 L within 24 h), compared to moderate intravenous rehydration, may result in faster hemodilution (hematocrit < 35%), a higher incidence of organ failure (16.5% vs. 4.9% and 7.6%, *p* = 0.013), an increased risk of sepsis, and reduced survival rate [19]. The rehydration volume and rehydration rate differ for aggressive intravenous fluid resuscitation. However, similarly, a study by Enrique de-Madaria, et al. found that rehydration of more than 4.1 L during the first 24 h of treatment for acute pancreatitis had a significant independent association with persistent organ failure, AKI, respiratory function and renal insufficiency [20]. A retrospective cohort study by Julton Tomanguillo, et al. based on the TriNetX Research Network showed that 24-hour aggressive intravenous rehydration (> 3 ml/kg/hr) was not found to be superior to non-aggressive intravenous rehydration in reducing the length of hospitalization and the risk of 30-day mortality, AKI, and sepsis in those with acute pancreatitis (≤ 1.5 ml/kg/hr) [21]. These findings suggest that during fluid resuscitation in SAP, individualized assessment should be performed to adjust the total amount of fluid and the rate of infusion at the early stage, because both insufficient and overexpanded plasma volume may have a negative impact on prognosis [22]. Prospective studies are warranted to further confirm whether ePVS, as an indicator for noninvasive, rapid and simple assessment of plasma volume, can be used as an additional indicator to assist in guiding early fluid resuscitation.

However, there are some limitations in the present study. Due to database limitations, we performed rigorous data preprocessing (excluding records with missing or contradictory critical variables), employed the modular approach adopted by the MIMIC-IV for data organization (e.g., ICD-9 coding and ICD-10 coding), and used multiple imputation to account for potential missing data. Additionally, we were unable to compare ePVS with traditional scoring systems (APACHE II, BISAP, etc.) due to incomplete data required for these systems. Although potential confounders were adjusted for during data collection and analysis, there may still be some bias that cannot be completely eliminated. Selection bias might arise from the specific inclusion criteria used in our study, potentially limiting the generalizability of our findings. Measurement bias could occur due to variations in how data were collected across different time points, which may affect the accuracy of ePVS estimates. It is worth noting that ePVS is not equivalent to actual plasma volume, and may be limited in specificity as a standalone predictor due to potential influences from factors such as anemia or dehydration. Therefore, more precise indicators for plasma volume are needed to further validate our findings. The Strauss formula provides a direct correlation among hematocrit, hemoglobin, and plasma volume. However, this easy tool may not fully encapsulate a complex dynamic change in plasma volume in critically ill patients. Therefore, further validation and refinement of the Strauss formula are warranted within the context of the SAP. Based on the results of our study, larger multicenter studies or independent cohort studies can be carried out in the future to enhance the external validity of the results and account for regional or institutional differences, such as variations in clinical protocols or socioeconomic factors. Moreover, our study focused on SAP patients. Therefore, our findings may not be applicable to non-SAP patients or SAP patients who were not admitted to the ICU. Future research is needed to validate our results in a broader population. Another limitation of this study is the absence of longitudinal tracking of ePVS, which precluded our ability to characterize dynamic associations between plasma volume fluctuations and clinical outcomes. Future prospective studies should prioritize serial ePVS measurements alongside detailed fluid management and clinical intervention records to elucidate how evolving plasma volume status interacts with treatment strategies and influences prognosis in critically ill patients.

## Conclusion

This study revealed a significant positive correlation of a higher ePVS value with the risk of 30-day ACM in the patients. This finding provides physicians with a more precise tool to assess the risk of 30-day ACM in critically ill patients at an early stage. By identifying high-risk patients, physicians can take timely and targeted therapeutic measures, which may significantly improve patient prognosis.

## Electronic supplementary material

Below is the link to the electronic supplementary material.

Supplementary Material 1

## Data Availability

The original contributions presented in the study are included in the article, further inquiries can be directed to the corresponding author.

## Abbreviations

SAP: Severe acute pancreatitis
ePVS: Estimated plasma volume status
ACM: All-cause mortality
RCS: Restricted cubic spline
AP: Acute pancreatitis
APACHE: Acute physiology and chronic health evaluation
BISAP: Bedside index of severity in acute pancreatitis
HT: Hypertension
AKI: Acute kidney injury
AF: Atrial fibrillation
DM: Diabetes mellitu
SOFA: Sequential organ failure assessment
HR: Heart rate
SBP: Systolic blood pressure
DBP: Diastolic blood pressure
MAP: Mean arterial pressure
RR: Respiratory rate
SPO2: Pulse oxygen saturation
ALP: Alkaline phosphatase
ALT: Alanine aminotransferase
AST: Aspartate aminotransferase
LDH: Lactate dehydrogenase
TBIL: Total bilirubin
BUN: Blood urea nitrogen
INR: International normalized ratio
Lac: Lactate
AG: Anion gap
WBC: White blood cell
PLT: Platelet
HCT: Hematocrit
MV: Mechanical ventilation
PPI: Proton pump inhibitors

## References

[1] Zerem E, Kurtcehajic A, Kunosić S, Zerem Malkočević D, Zerem O. Current trends in acute pancreatitis: diagnostic and therapeutic challenges. World J Gastroenterol. 2023;29(18):2747–63. 37274068 10.3748/wjg.v29.i18.2747 PMC10237108

[2] Heckler M, Hackert T, Hu K, Halloran CM, Büchler MW, Neoptolemos JP. Severe acute pancreatitis: surgical indications and treatment. Langenbecks Arch Surg. 2021;406(3):521–35. 32910276 10.1007/s00423-020-01944-6 PMC8106572

[3] Huang Y, Badurdeen DS. Acute pancreatitis review. Turk J Gastroenterol. 2023;34(8):795–801. 37404118 10.5152/tjg.2023.23175 PMC10544623

[4] Mederos MA, Reber HA, Girgis MD. Acute pancreatitis: A review. JAMA. 2021;325(4):382–90. 33496779 10.1001/jama.2020.20317

[5] Singh VK, Wu BU, Bollen TL, Repas K, Maurer R, Johannes RS, Mortele KJ, Conwell DL, Banks PA. A prospective evaluation of the bedside index for severity in acute pancreatitis score in assessing mortality and intermediate markers of severity in acute pancreatitis. Am J Gastroenterol. 2009;104(4):966–71. 19293787 10.1038/ajg.2009.28

[6] Sahu B, Abbey P, Anand R, Kumar A, Tomer S, Malik E. Severity assessment of acute pancreatitis using CT severity index and modified CT severity index: correlation with clinical outcomes and severity grading as per the revised Atlanta classification. Indian J Radiol Imaging. 2017;27(2):152–60. 28744075 10.4103/ijri.IJRI_300_16 PMC5510312

[7] Duarte K, Monnez JM, Albuisson E, Pitt B, Zannad F, Rossignol P. Prognostic value of estimated plasma volume in heart failure. JACC Heart Fail. 2015;3(11):886–93. 26541787 10.1016/j.jchf.2015.06.014

[8] Chen J, Shen J, Cai D, Wei T, Qian R, Zeng C, Lyu L. Estimated plasma volume status (ePVS) is a predictor for acute myocardial infarction in-hospital mortality: analysis based on MIMIC-III database. BMC Cardiovasc Disord. 2021;21(1):530. 34749646 10.1186/s12872-021-02338-2 PMC8573972

[9] Nogi K, Ueda T, Nakamura T, Nogi M, Ishihara S, Nakada Y, Hashimoto Y, Nakagawa H, Nishida T, Seno A, et al. New classification for the combined assessment of the fractional excretion of Urea nitrogen and estimated plasma volume status in acute heart failure. J Am Heart Assoc. 2023;12(1):e025596. 36583422 10.1161/JAHA.122.025596 PMC9973588

[10] Yogeswaran A, Richter MJ, Husain-Syed F, Rako Z, Sommer N, Grimminger F, Seeger W, Ghofrani HA, Gall H, Tello K. Estimated plasma volume status: association with congestion, cardiorenal syndrome and prognosis in precapillary pulmonary hypertension. Front Cardiovasc Med. 2023;10:1161041. 37234373 10.3389/fcvm.2023.1161041 PMC10206211

[11] Turcato G, Zaboli A, Ciccariello L, Pfeifer N. Estimated plasma volume status (ePVS) could be an easy-to-use clinical tool to determine the risk of sepsis or death in patients with fever. J Crit Care. 2020;58:106–12. 32422322 10.1016/j.jcrc.2020.05.001

[12] Abidin ZU, Naqvi RA, Haider A, Kim HS, Jeong D, Lee SW. Recent deep learning-based brain tumor segmentation models using multi-modality magnetic resonance imaging: a prospective survey. Front Bioeng Biotechnol. 2024;12:1392807. 39104626 10.3389/fbioe.2024.1392807 PMC11298476

[13] Imran SMA, Saleem MW, Hameed MT, Hussain A, Naqvi RA, Lee SW. Feature preserving mesh network for semantic segmentation of retinal vasculature to support ophthalmic disease analysis. Front Med (Lausanne). 2022;9:1040562. 36714120 10.3389/fmed.2022.1040562 PMC9880050

[14] de-Madaria E, Buxbaum JL, Maisonneuve P, García García de Paredes A, Zapater P, Guilabert L, Vaillo-Rocamora A, Rodríguez-Gandía M, Donate-Ortega J, Lozada-Hernández EE, et al. Aggressive or moderate fluid resuscitation in acute pancreatitis. N Engl J Med. 2022;387(11):989–1000. 36103415 10.1056/NEJMoa2202884

[15] Johnson AEW, Bulgarelli L, Shen L, Gayles A, Shammout A, Horng S, Pollard TJ, Hao S, Moody B, Gow B, et al. MIMIC-IV, a freely accessible electronic health record dataset. Sci Data. 2023;10(1):1. 36596836 10.1038/s41597-022-01899-x PMC9810617

[16] Strauss MB, Davis RK, Rosenbaum JD, Rossmeisl EC. Water diuresis produced during recumbency by the intravenous infusion of isotonic saline solution. J Clin Invest. 1951;30(8):862–8. 14861307 10.1172/JCI102501 PMC436321

[17] Lucijanic M, Krecak I, Soric E, Sabljic A, Galusic D, Holik H, Perisa V, Peric MM, Zekanovic I, Kusec R. Higher estimated plasma volume status is associated with increased thrombotic risk and impaired survival in patients with primary myelofibrosis. Biochem Med (Zagreb). 2023;33(2):020901. 37143717 10.11613/BM.2023.020901 PMC10152616

[18] Zerem E. Treatment of severe acute pancreatitis and its complications. World J Gastroenterol. 2014;20(38):13879–92. 25320523 10.3748/wjg.v20.i38.13879 PMC4194569

[19] Messallam AA, Body CB, Berger S, Sakaria SS, Chawla S. Impact of early aggressive fluid resuscitation in acute pancreatitis. Pancreatology. 2021;21(1):69–73. 33257225 10.1016/j.pan.2020.11.006

[20] de-Madaria E, Soler-Sala G, Sánchez-Payá J, Lopez-Font I, Martínez J, Gómez-Escolar L, Sempere L, Sánchez-Fortún C, Pérez-Mateo M. Influence of fluid therapy on the prognosis of acute pancreatitis: a prospective cohort study. Am J Gastroenterol. 2011;106(10):1843–50. 21876561 10.1038/ajg.2011.236

[21] Tomanguillo J, Searls L, Annie FH, Kemper S, Drabish K, Naravadi V. A nationwide analysis of fluid resuscitation outcomes in patients with acute pancreatitis. Cureus. 2023;15(12):e50182. 38192944 10.7759/cureus.50182 PMC10771961

[22] Lucijanic M, Krecak I, Busic I, Atic A, Stojic J, Sabljic A, Soric E, Veic P, Marevic S, Derek L, et al. Estimated plasma volume status in COVID-19 patients and its relation to comorbidities and clinical outcomes. J Thromb Thrombolysis. 2024;57(1):50–7. 37572182 10.1007/s11239-023-02882-y

