# Supplementary material for: Association between estimated plasma volume status and the risk of 30-day mortality in patients with severe acute pancreatitis: a retrospective study based on the MIMIC-IV database
Source: BMC Gastroenterol. 2025 Apr 29;25:314. doi: 10.1186/s12876-025-03895-y (PMC12042456; doi:10.1186/s12876-025-03895-y)
Supplement: Supplementary file 1 — Supplementary Material 1 [file 12876_2025_3895_MOESM1_ESM.doc]

**STROBE Statement**

Checklist of items that should be included in reports of observational studies

| **Section/Topic** | Item No | Recommendation | Reported on Page No |
| --- | --- | --- | --- |
| **Title and abstract** | 1 | (*a*) Retrospective Cohort Study |  |
|  |  | (*b*) The study aimed to investigate the relationship between estimated plasma volume status (ePVS) and the risk of 30-day all-cause mortality (ACM) in patients with severe acute pancreatitis (SAP).The study's findings indicate a significant association between high ePVS values and an increased risk of 30-day ACM in SAP patients. | 1 |
| Introduction | | | |
| Background/rationale | 2 | Plasma volume status (PVS) is an important physiological parameter that reflects a patient's hydration and circulatory status.Plasma volume changes can be a prognostic indicator in critically ill patients. In SAP, alterations in plasma volume may reflect the severity of the disease and the patient's response to treatment.Despite the importance of plasma volume assessment, the relationship between estimated plasma volume status (ePVS) and the prognosis of SAP, particularly in terms of mortality, has not been well established. This gap in knowledge motivated the researchers to conduct a study aimed at elucidating this relationship. | 3 |
| Objectives | 3 | The primary objective was to assess the association between ePVS and the risk of 30-day all-cause mortality in SAP patients. | 4 |
| Methods | | | |
| Study design | 4 | Identifies the study as a retrospective cohort study using data from the MIMIC-IV database. | 4 |
| Setting | 5 | The study setting is the Beth Israel Deaconess Medical Center in Boston, and the data used spans from 2008 to 2019. The recruitment and exposure of patients occurred during their ICU stay within this period, with a 30-day follow-up for the study's primary outcome measurement. The exact dates for data extraction and analysis are presumed to be after the author's certification for accessing the MIMIC-IV database. | 5 |
| Participants | 6 | (*a*)The study included adult ICU patients with AP, excluded those with missing hematocrit or hemoglobin data, and followed them for 30 days post-hospitalization to determine mortality outcomes. The follow-up was conducted through the analysis of data extracted from the MIMIC-IV database, without direct patient contact, as it is a retrospective study based on existing clinical data. | 5 |
|  |  | (*b*) This study is not designed as a matched study. Instead, it analyzes data from a cohort of patients with SAP, stratified by ePVS quartiles, to assess the risk of 30-day ACM. |  |
| Variables | 7 | Primary Outcome:30-day All-Cause Mortality (ACM):The risk of death from any cause within 30 days from the first day of hospitalization for patients with severe acute pancreatitis (SAP) admitted to the ICU.Estimated Plasma Volume Status (ePVS):The main exposure of interest, which is a calculated measure derived from hematocrit and hemoglobin levels. It serves as a predictor for the primary outcome.  Predictors:1. ePVS as a Continuous Variable:The direct relationship between the level of ePVS and the risk of 30-day ACM.  2. ePVS as a Categorical Variable:The study also examines ePVS divided into quartiles (Q1 to Q4) to assess the risk of 30-day ACM across different levels of ePVS.  Potential Confounders:The study adjusts for several potential confounders using multivariate Cox regression analysis. These include:Demographic Factors(Age,Gender,Race, Marital status), Comorbidities( Obesity and overweight, hypertension (HT), acute kidney injury (AKI), atrial fibrillation (AF), diabetes mellitus (DM), heart failure),Scoring Systems( Sequential Organ Failure Assessment (SOFA) score, Severe Acute Pancreatitis Score II (SAPS II)), Vital Signs(Heart rate (HR),Systolic blood pressure (SBP),Diastolic blood pressure (DBP),Mean arterial pressure (MAP),Respiratory rate (RR), Pulse oxygen saturation (SPO2),Temperature), Laboratory Results(Albumin,Alkaline phosphatase (ALP),Alanine aminotransferase (ALT),Aspartate aminotransferase (AST),Lactate dehydrogenase (LDH),Total bilirubin (TBIL),Blood urea nitrogen (BUN),Creatinine,Blood lipase,Phosphate,Glucose,Potassium,Sodium,Total calcium,International normalized ratio (INR),Lactate (Lac),Anion gap (AG),Bicarbonate,White blood cell (WBC) count,Platelet (PLT) count,Hematocrit (HCT),Hemoglobin (HBG)), Treatments(Mechanical ventilation (MV),Use of norepinephrine,Use of statins,Use of proton pump inhibitors (PPIs), Use of octreotide).  The study performs subgroup analyses to identify potential effect modifiers, which include:Age( ≥60 vs. <60 years), Gender,Marital Status,Race,AKI,AF,DM,Heart Failure,Obesity and Overweight,HT,Ventilation,Norepinephrine Use,Statins Use,PPIs Use,Octreotide Use. | 5 |
| Data sources/measurement | 8* | Source: MIMIC-IV database.  Assessment: Extracted directly from patient records within the database. | 5 |
| Bias | 9 | 1.Leveraging a Large Database:By utilizing the MIMIC-IV database, which contains extensive clinical data from patients at Beth Israel Deaconess Medical Center, the study aims to enhance the representativeness and statistical power of the findings.  2. Strict Patient Selection Criteria: The study clearly defines inclusion and exclusion criteria to ensure homogeneity among study subjects and to minimize selection bias.  3. Data Cleaning and Imputation:For missing data, the study employs multiple imputation methods to handle less than 20% missing data, reducing bias that could result from data absence.  4. Outlier Management: The Winsorization method is used to address outliers, mitigating their impact on data analysis.  5. LASSO Regression for Covariate Selection: LASSO regression with 10-fold cross-validation is used to identify covariates associated with 30-day all-cause mortality, helping to prevent overfitting and bias in variable selection.  6. Multivariate Cox Proportional Hazards Model:The study attempts to control for the influence of multiple potential confounders by adjusting them in the Cox regression model, thereby reducing confounding bias.  7. Restricted Cubic Spline (RCS) Analysis:RCS analysis is used to assess the nonlinear relationship between ePVS and 30-day all-cause mortality, allowing for a more accurate depiction of the relationship between variables.  8. Kaplan-Meier Survival Curves and Log-Rank Test: Kaplan-Meier survival curves are used to compare survival probabilities across different ePVS groups, and the log-rank test is employed to compare survival differences, standard statistical methods to minimize bias in survival analysis.  9. Subgroup Analysis: Subgroup analyses are conducted to evaluate the relationship between ePVS and 30-day all-cause mortality within different subgroups, identifying potential effect modifiers and assessing the consistency of results across various populations.  10. Statistical Significance Level:A two-tailed p-value of less than 0.05 is set as the standard for statistical significance to reduce the risk of Type I error (incorrectly rejecting a true null hypothesis). | 6 |
| Study size | 10 | The study size was determined by the intersection of the database population, the application of inclusion and exclusion criteria and the completeness of the required data,. The final study size of 1036 patients was the result of these factors. | 6 |
| Quantitative variables | 11 | 1. Grouping of Quantitative Variables:  - Quartiles: The estimated plasma volume status (ePVS), a key quantitative variable, was divided into quartiles (Q1 to Q4) to assess the risk of 30-day all-cause mortality (ACM) across different levels of ePVS. This non-parametric grouping method helps to understand the distribution of ePVS in the population and to identify any potential threshold effects.  - Cut-off Value:A cut-off value of 6.23 dL/g for ePVS was determined using Restricted Cubic Spline (RCS) analysis. This cut-off value was used to dichotomize the continuous variable into a binary variable, which can simplify the interpretation of the results and identify a potential threshold above which the risk of ACM increases.  2. Statistical Modeling:  - LASSO Regression: Quantitative variables with less than 20% missing data were included in the Least Absolute Shrinkage and Selection Operator (LASSO) regression analysis to screen for relevant covariates. This method helps to reduce the dimensionality of the data and to select a subset of variables that are most strongly associated with the outcome.  - Cox Proportional Hazards Model: Both continuous and categorical forms of ePVS were included in the Cox regression models to assess the association between ePVS and the risk of 30-day ACM. The continuous variable allows for a more nuanced understanding of the relationship, while the categorical variable simplifies the interpretation of the results.  3. Handling of Missing Data:  - Multiple Imputation: Quantitative variables with less than 20% missing data were handled using multiple imputation, a method that creates several plausible imputed values for each missing value, reflecting the uncertainty about the right value to impute. This approach helps to reduce bias and increase the precision of the estimates compared to simpler methods like complete case analysis.  4. Transformation and Normality Checks:  - Winsorization: To reduce the impact of outliers on the analysis, the study used the Winsorization method, which involves capping extreme values at a certain percentile (e.g., 99% and 1%). This transformation helps to stabilize the variance and reduce the influence of outliers.  - Normality Assessment: Continuous variables were checked for normality using the Shapiro-Wilks test. Variables that followed a normal distribution were analyzed using parametric tests (e.g., t-test), while non-normally distributed variables were expressed as medians (interquartile range) and analyzed using nonparametric methods (e.g., Mann-Whitney U test). | 6 |
| Statistical methods | 12 | (*a*) LASSO Regression: This method was used to screen for relevant covariates that are associated with the risk of 30-day ACM. LASSO (Least Absolute Shrinkage and Selection Operator) regression is a type of penalized regression that can shrink some coefficients to zero, effectively performing variable selection. The manuscript mentions the use of 10-fold cross-validation to determine the optimal value of the regularization parameter λ.  Restricted Cubic Spline (RCS) Analysis: This non-linear regression technique was used to assess the non-linear relationship between ePVS and the risk of 30-day ACM. RCS can flexibly model the shape of the relationship between a continuous predictor and an outcome, allowing for potential non-linear patterns.  Kaplan-Meier Survival Curve: This method was used to estimate the survival function and to compare the 30-day survival across different groups defined by quartiles of ePVS. The log-rank test was used to compare survival curves between groups.  Cox Regression Analysis: This is a semi-parametric model used for survival analysis. The manuscript describes both univariate and multivariate Cox regression models to assess the association between ePVS and the risk of 30-day ACM.  Model I (Unadjusted): This model did not include any covariates, providing an unadjusted estimate of the hazard ratio (HR) for ePVS.  Model II: Adjusted for age.  Model III: Further adjusted for a range of covariates including systolic blood pressure (SBP), albumin, blood urea nitrogen (BUN), international normalized ratio (INR), lactate dehydrogenase (LDH), blood lipase, total bilirubin (TBIL), acute kidney injury (AKI), diabetes mellitus (DM), hyperlipidemia, norepinephrine use, and statin use. These covariates were selected to control for potential confounding factors that could influence the relationship between ePVS and 30-day ACM. | 6 |
|  |  | (*b*)Subgroup analysis was conducted to explore whether the association between estimated plasma volume status (ePVS) and the risk of 30-day all-cause mortality (ACM) varies across different subgroups.The defined subgroups included age (≥60 years vs. <60 years), gender, marital status, race, acute kidney injury (AKI), atrial fibrillation (AF), diabetes mellitus (DM), heart failure, obesity and overweight, hypertension (HT), use of norepinephrine, statins, proton pump inhibitors (PPIs), octreotide, and mechanical ventilation (MV).The subgroup analysis revealed significant associations within specific subgroups, such as patients younger than 60 years (HR=1.25, 95% CI: 1.07-1.45), white patients (HR=1.15, 95% CI: 1.03-1.28), and patients experiencing AKI (HR=1.11, 95% CI:1.02-1.21).Subgroup analysis was performed by including interaction terms between ePVS and subgroup variables in the Cox regression models. | 6 |
|  |  | (*c*) Exclusion of Variables with High Missing Data:Variables with more than 20% missing data were excluded from the analysis. This is a common practice to prevent the introduction of significant bias that could be caused by a large proportion of missing values for a particular variable.  Multiple Imputation for Variables with Less Missing Data:For variables that had less than 20% missing data, multiple imputation was used to estimate the missing values. Multiple imputation is a statistical technique that involves creating several plausible values for the missing data points, based on the observed data. This method helps to reduce bias and increases the precision of the estimates by considering the uncertainty around the missing data. | 6 |
|  |  | (*d*) The study is a retrospective analysis based on the MIMIC-IV database, which contains de-identified clinical data from patients who were hospitalized at the Beth Israel Deaconess Medical Center between 2008 and 2019. In such retrospective database studies, there is typically no follow-up of patients after discharge, and the data available for analysis are limited to what has been recorded in the medical records during the hospital stay. | 6 |
|  |  |  |  |

| **Section/Topic** | Item No | Recommendation | Reported on Page No |
| --- | --- | --- | --- |
| Results | | | |
| Participants | 13* | (a) Patients aged over 18 years and initially admitted to the ICU were included. The exclusion criteria were non-ICU patients and patients with missing data on hematocrit or hemoglobin.The final number of patients confirmed eligible and included in the study was 1,036.Since this is a retrospective database study, there was no active follow-up of patients. The data available for analysis were limited to what was recorded during the patients' hospital stay.All 1,036 included patients were analyzed in the study. The manuscript mentions that there were 899 (86.8%) 30-day survivors and 137 (13.2%) deaths. | 7 |
|  |  | (b) In this retrospective database studies, the concept of follow-up is different from that in prospective studies. The data analysis is conducted on the available records, and there is no loss to follow-up in the traditional sense because the analysis is based on the complete dataset as it exists in the database at the time of the study. | 7 |
|  |  | (c) 1.Patients in MIMIC-IV Database: This would be the starting point, representing all patients in the MIMIC-IV database that could potentially be included in the study.  2.Patients with AP Diagnosis: This stage would represent the filtering process where patients are identified based on ICD-9-CM and ICD-10-CM codes for acute pancreatitis.  3.After Applying Inclusion/Exclusion: Here, the flow diagram would show the application of specific inclusion criteria (e.g., age > 18 years, admitted to the ICU) and exclusion criteria (e.g., non-ICU patients, missing data on hematocrit or hemoglobin). This step would lead to the final number of patients included in the analysis.  4.Final Analysis: The final stage would indicate the number of patients who were actually analyzed in the study, which in this case is 1,036 patients. | 8 |
| Descriptive data | 14* | (a) 1.Demographic Characteristics:  Age: The study included patients both younger than 60 years (50.6%) and 60 years or older (49.4%).  Gender: There were 583 males (56.3%) and 453 females (43.7%) in the study.  Race: The majority of the patients were white (65.3%), followed by non-white (25.3%), and unknown (9.46%).  Marital Status: Included single (32.9%), divorced/widowed (17.9%), married (43.1%), and unknown (6.18%).  Clinical Characteristics:  2.Comorbidities: Patients had various comorbidities such as obesity and overweight, hypertension, acute kidney injury, atrial fibrillation, diabetes mellitus, and heart failure.  3.Scoring Systems: Patients were assessed using the Sequential Organ Failure Assessment (SOFA) score and Severe Acute Pancreatitis Score II (SAPS II).  4.Vital Signs: Information on heart rate, systolic and diastolic blood pressure, mean arterial pressure, respiratory rate, pulse oxygen saturation, and temperature was collected.  5.Laboratory Results: Data included albumin, alkaline phosphatase, alanine aminotransferase, aspartate aminotransferase, lactate dehydrogenase, total bilirubin, blood urea nitrogen, creatinine, blood lipase, and other lab values.  Social Characteristics:  6.Exposures:The primary exposure of interest in this study is the estimated plasma volume status (ePVS), which is calculated using the Strauss formula based on hematocrit and hemoglobin levels.  7.Potential Confounders:The study used LASSO regression to identify covariates linked to the risk of 30-day ACM. These covariates, which serve as potential confounders, include age, systolic blood pressure (SBP), albumin, blood urea nitrogen (BUN), international normalized ratio (INR), lactate dehydrogenase (LDH), blood lipase, total bilirubin (TBIL), acute kidney injury (AKI), diabetes mellitus (DM), hyperlipidemia, norepinephrine use, and statin use.These confounders were adjusted for in the multivariate Cox regression models to assess their impact on the relationship between ePVS and the risk of 30-day ACM. | 8 |
|  |  | (b)Variables with More than 20% Missing Data:These variables were excluded from the analysis. The exact number of variables and the specific variables excluded due to high percentages of missing data are not specified in the document.  Variables with Less than 20% Missing Data:These variables were interpolated using a multiple imputation method. | 8 |
|  |  | (c) In the context of this study, "follow-up" refers to the observation period within which the primary outcome (30-day ACM) was assessed. There is no additional follow-up beyond the 30-day mark, and the study does not track patients beyond their hospital stay. The data used for the analysis are static, reflecting the information documented in the medical records up to the 30-day mark or the time of discharge or death, whichever came first. | 8 |
| Outcome data | 15* | Total Number of Patients: The study included a total of 1,036 patients.  Survival Rate: The 30-day survival rate was 86.8%, which means that 899 out of 1,036 patients survived within the 30-day period following their hospital admission.  Mortality Rate: The 30-day mortality rate was 13.2%, with 137 out of 1,036 patients dying within the 30-day period.  Mortality by ePVS Quartiles:  Q1 group (lowest ePVS): 9.69% mortality (25 out of 258 patients).  Q2 group: 9.62% mortality (25 out of 260 patients).  Q3 group: 15.8% mortality (41 out of 259 patients).  Q4 group (highest ePVS): 17.8% mortality (46 out of 259 patients). | 9 |
|  |  | *Case-control study—*Report numbers in each exposure category, or summary measures of exposure | 9 |
|  |  | *Cross-sectional study—*Report numbers of outcome events or summary measures | 9 |
| Main results | 16 | (*a*) 1.Unadjusted Estimates (Model I):  ePVS as a Continuous Variable:HR (Hazard Ratio) = 1.16, 95% CI (Confidence Interval): 1.07 - 1.26, p-value < 0.001  ePVS as a Categorical Variable (Q4 vs. Q1):HR = 1.94, 95% CI: 1.19 - 3.16, p-value = 0.008  2.Confounder-Adjusted Estimates (Model III):  ePVS as a Continuous Variable:HR = 1.09, 95% CI: 1.01 - 1.18, p-value = 0.035  ePVS as a Categorical Variable (Q4 vs. Q1):, HR = 1.70, 95% CI: 1.03 - 2.80, p-value = 0.039  3.Confounders Adjusted For:  The confounders adjusted in Model III include:Age, Systolic blood pressure (SBP), Albumin,Blood urea nitrogen (BUN), International normalized ratio (INR), Lactate dehydrogenase (LDH), Blood lipase, Total bilirubin (TBIL), Acute kidney injury (AKI), Diabetes mellitus (DM), Hyperlipidemia, Use of norepinephrine, Use of statins,.  4.Rationale for Including Confounders:These confounders were selected because they are clinically relevant and have the potential to influence the relationship between ePVS and 30-day ACM. They represent a range of physiological, biochemical, and treatment-related factors that could affect patient outcomes in severe acute pancreatitis. By adjusting for these variables, the study aims to isolate the effect of ePVS on mortality while accounting for other factors that could confound the relationship. | 9 |
|  |  | (*b*) The categorization of the estimated plasma volume status (ePVS) into quartiles. The boundaries for these quartiles are as follows:Q1 Group (Lowest ePVS):ePVS ≤ 5.14 dL/g; Q2 Group:5.14 dL/g < ePVS ≤ 6.26 dL/g; Q3 Group:6.26 dL/g < ePVS ≤ 7.77 dL/g; Q4 Group (Highest ePVS):ePVS > 7.77 dL/g | 9 |
|  |  | (*c*) The study provides estimates of relative risk in the form of hazard ratios (HRs) for the association between estimated plasma volume status (ePVS) and the risk of 30-day all-cause mortality (ACM). To translate these relative risk estimates into absolute risk, we need to consider the baseline risk in the population and the time period of interest, which is 30 days in this case.  Baseline Risk:  The overall 30-day survival rate in the study was 86.8%, which means the baseline 30-day mortality rate is: 1−0.868=0.132 or 13.2%1−0.868=0.132 or 13.2%  Absolute Risk Calculation:  Absolute risk can be calculated by multiplying the baseline risk by the hazard ratio (HR) for each category of ePVS. However, since the HRs provided are relative measures, we need to convert them into absolute differences in risk for the 30-day period.  For ePVS as a Continuous Variable (HR = 1.09):  The HR of 1.09 suggests that for every one-unit increase in ePVS (dL/g), the risk of 30-day ACM increases by 9%.  To find the absolute risk difference, we can use the formula: Absolute Risk Increase=Baseline Risk×(HR−1)Absolute Risk Increase=Baseline Risk×(HR−1)  For a one-unit increase in ePVS: 0.132×(1.09−1)=0.132×0.09=0.01188 or 1.188%0.132×(1.09−1)=0.132×0.09=0.01188 or 1.188%  For ePVS as a Categorical Variable (Q4 vs. Q1, HR = 1.70):  The HR of 1.70 for the Q4 group compared to the Q1 group suggests that patients in the Q4 group have a 70% higher risk of 30-day ACM than those in the Q1 group.  To find the absolute risk difference: Absolute Risk Increase=Baseline Risk×(HR−1)Absolute Risk Increase=Baseline Risk×(HR−1)  For Q4 vs. Q1: 0.132×(1.70−1)=0.132×0.70=0.0924 or 9.24%0.132×(1.70−1)=0.132×0.70=0.0924 or 9.24% | 9 |
| Other analyses | 17 | Subgroup analyses were performed to assess whether the association between ePVS and 30-day ACM varied across different demographic and clinical subgroups. The subgroups included:Age (≥60 vs. <60 years), Gender, Marital status, Race, Acute kidney injury (AKI), Atrial fibrillation (AF), Diabetes mellitus (DM), Heart failure, Obesity and overweight, Hypertension (HT), Use of norepinephrine, Use of statins, Use of proton pump inhibitors (PPIs), Use of octreotide, Mechanical ventilation (MV).  The results revealed significant associations within specific subgroups, indicating that the relationship between ePVS and ACM risk might differ based on these characteristics. | 9 |
| Discussion | | | |
| Key results | 18 | The key results of the study are be summarized as follows:  1. Patient Cohort and Survival Rate:  - The study included 1,036 patients with SAP admitted to the ICU.  - The 30-day survival rate was 86.8%, with 899 patients surviving and 137 patients dying within 30 days.  2. ePVS and Mortality Risk:  - Patients were divided into four groups based on quartiles of ePVS.  - The Kaplan-Meier survival curve showed that the high ePVS group (Q4) was at a higher risk of 30-day ACM (p=0.007).  3. Unadjusted and Adjusted Estimates:  - Unadjusted analysis showed a positive association of ePVS as a continuous variable with the risk of 30-day ACM (HR=1.16, 95% CI: 1.07-1.26, p < 0.001).  - After adjusting for confounders (Model III), ePVS remained significantly associated with the risk of 30-day ACM as a continuous variable (HR=1.09, 95% CI: 1.01-1.18, p=0.035).  4. Categorical Analysis:  - When ePVS was treated as a categorical variable, the Q4 group had a higher risk of 30-day ACM compared to the Q1 group in both unadjusted (HR=1.94, 95% CI: 1.19-3.16, p=0.008) and adjusted (HR=1.70, 95% CI: 1.03-2.80, p=0.039) analyses.  5. Subgroup Analyses:  - Subgroup analyses revealed significant associations between ePVS and 30-day ACM within specific subgroups, such as patients younger than 60 years, white patients, patients with AKI, and patients with comorbid DM.  6. Interaction Analyses:  - No significant interactions were found between ePVS and any of the subgroup variables, indicating that the association between ePVS and ACM risk is consistent across different patient characteristics. | 10 |
| Limitations | 19 | Limitations:  1. Retrospective Design:  - The study is based on a retrospective analysis of the MIMIC-IV database, which may introduce biases due to the reliance on existing data that was not collected specifically for this study. This could lead to missing data or data that are not uniformly collected.  2. Missing Data:  - While the study used multiple imputation to handle missing data, this method relies on assumptions about the missingness mechanism. If these assumptions are violated, the imputation could introduce bias.  3. Confounding:  - Although the study adjusted for several potential confounders, there may be residual confounding from unmeasured or unknown factors that were not included in the analysis.  4. ePVS as a Surrogate Measure:  - ePVS is not equivalent to actual plasma volume, and its use as a surrogate measure could introduce some imprecision in the estimation of the true relationship between plasma volume and mortality.  5. Generalizability:  - The study was conducted using data from a single center, which may limit the generalizability of the findings to other populations or settings.  6. Data Quality:  - The quality of the data depends on the accuracy and completeness of the medical records in the MIMIC-IV database. Errors or inconsistencies in data entry could affect the results.  Potential Bias and Imprecision:  1. Selection Bias:  - The study's patient population may not be representative of all patients with SAP, which could lead to selection bias. The direction of this bias would depend on the characteristics of the patients included in the database compared to the broader population of SAP patients.  2. Information Bias:  - There could be information bias if the data on ePVS or other variables were recorded inaccurately or inconsistently. This bias could be in either direction, depending on whether the errors led to overestimation or underestimation of ePVS. | 13 |
| Interpretation | 20 | The study provides evidence suggesting that higher ePVS values are associated with an increased risk of 30-day ACM in SAP patients. This association appears to be robust to adjustments for potential confounding variables and is consistent across various patient subgroups. However, given the limitations and the multiplicity of analyses conducted, these findings should be interpreted cautiously. The results support the need for further prospective studies with robust data collection to confirm the relationship between ePVS and mortality, and to explore the causal pathways involved. Until then, while ePVS shows promise as a prognostic marker, its use in clinical decision-making should be considered experimental, and treatment strategies should not be based solely on ePVS without further validation. | 14 |
| Generalisability | 21 | While the study provides valuable insights into the relationship between ePVS and 30-day ACM in SAP patients, its generalizability is limited by its single-center design, reliance on a specific database, and focus on ICU patients. To enhance the external validity of the findings, future studies should consider:  1.Conducting multicenter trials to capture a more diverse patient population and different treatment practices.  2.Including patients from various geographical regions and healthcare systems to assess the consistency of the findings across different settings.  3.Conducting prospective studies with standardized protocols to minimize biases and improve the quality of evidence.  Until such studies are conducted, the findings should be applied cautiously, and their relevance to other populations or settings should be critically evaluated. | 14 |
| Other Information | | | |
| Funding | 22 | This study was conducted without any external funding, and therefore, there were no funders involved in the study. | 16 |

**Give information separately for cases and controls in case-control studies and, if applicable, for exposed and unexposed groups in cohort and cross-sectional studies.*

**Note:** An Explanation and Elaboration article discusses each checklist item and gives methodological background and published examples of transparent reporting. The STROBE checklist is best used in conjunction with this article (freely available on the Web sites of PLoS Medicine at http://www.plosmedicine.org/, Annals of Internal Medicine at http://www.annals.org/, and Epidemiology at http://www.epidem.com/). Information on the STROBE Initiative is available at www.strobe-statement.org.
